# ESR as a monitoring method of the interactions between TEMPO-functionalized magnetic nanoparticles and yeast cells

**DOI:** 10.1038/s41598-019-55335-z

**Published:** 2019-12-10

**Authors:** Ryszard Krzyminiewski, Bernadeta Dobosz, Grzegorz Schroeder, Joanna Kurczewska

**Affiliations:** 1 Medical Physics Division, Faculty of Physics, Adam Mickiewicz University in Poznań, Uniwersytetu Poznańskiego 2, 61-614 Poznań, Poland; 2 Faculty of Chemistry, Adam Mickiewicz University in Poznań, Uniwersytetu Poznańskiego 8, 61-614, Poznań, Poland

**Keywords:** Biophysics, Nanoscience and technology

## Abstract

Potential application of magnetic nanoparticles as drug carriers in medical treatment requires prior determination of their effects on cells. In this work different spin labels and magnetic nanoparticles functionalized with spin labels as well as their interaction with yeast cells were investigated using electron spin resonance (ESR) method. ESR was demonstrated to be a suitable method for monitoring of magnetic core and attached spin labels. Particular emphasis was placed on characterization of endocytosis and redox processes running inside the cell, resulting in recombination of spin labels. Such data could only be obtained at reduced temperature of ESR measurements.

## Introduction

Nanomaterials are extensively studied for their use in various fields^1,2^. Biomedical applications of these materials, including new strategies in medical treatment and therapy^3–5^, are especially of great interest. Particularly promising is a potential role of nanoparticles as drug carriers^6,7^ in order to reduce therapeutic dose and deliver a drug directly to a desired location^8,9^. Nanomaterials with magnetic properties can be controlled by an appropriate magnetic field configuration^10,11^ that should facilitate targeted delivery of an attached drug.

The properties of magnetic nanoparticles are investigated using different methods^12^. However, penetration of nanoparticles into cellular structures and process of endocytosis are difficult to be monitored. Endocytosis is a complex phenomenon of cellular uptake of various particles^13–16^, during which plasma membrane invaginators to form vesicles that are essential for delivery of many substances into a cell. It can be also potentially applied for delivery of nanoparticles functionalized with drugs to cellular structures. Currently nanoparticles with attached fluorescent marker are used. They are characterized by confocal microscopy, as a direct technique of assessing the penetration of those nanoparticles inside the cell, and indirect biochemical methods^17–21^. Therefore, other methods to study endocytosis are currently searched.

Electron spin resonance (ESR) is commonly used for characterization of physical properties of various nanomaterials^22–28^, including functionalized magnetic nanoparticles^29,30^. The magnetic nanoparticles with attached spin label (free radical) are characterized by two areas that can be described by ESR measurements - magnetic core and free radical. Moreover, the method can be applied to observe the differences resulting from interaction between the material surface and environment^31,32^. Therefore, interaction of nanoparticles with cells, including their penetration into cells and the process of endocytosis, should be able to be monitored on the basis of ESR results.

The aim of this study was to show that electron spin resonance spectroscopy can be used not only to control the properties of functionalized magnetic nanoparticles but also to evaluate their interaction with model cells (yeast cells).

## Results

### Characterization of magnetic nanomaterials

Bare and coated magnetite nanoparticles were characterized by several physicochemical methods. All magnetic nanoparticles studied show bands in Fourier transform infrared (FTIR) spectra characteristic for iron(II, III) oxide (Fe_3_O_4_). Strong and broad band at 575 cm^−1^, observed in the spectrum of bare magnetite Fe_3_O_4_ and silica coated material Fe_3_O_4_@SiO_2_ (Fig. S1 in Supplementary information), is assigned to Fe-O vibration from the magnetite phase. The bands appearing at 3420 and 1639 cm^−1^ are related to -OH stretching vibrations (surface hydroxyl groups and water molecules). Silica coating is confirmed by a strong band at 1104 cm^−1^ (Si-O-Si asymmetric stretching vibration). Magnetic silica nanoparticles Fe_3_O_4_@SiO_2_ were further coated with dextran derivatives containing free radicals: 4-Amino-2,2,6,6-tetramethylpiperidine-1-oxyl (4-Amino-TEMPO) and 4-Hydroxy-2,2,6,6-tetramethylpiperidine-1-oxyl (TEMPOL), i.e. Dextran-NH-TEMPO and Dextran-O-TEMPO. However, the IR spectra of Fe_3_O_4_@SiO_2_@Dextran-NH-TEMPO and Fe_3_O_4_@SiO_2_@Dextran-O-TEMPO are very similar, and differ from the previous ones only by the presence of a new signal at 2935 cm^−1^ assigned to the C-H stretching vibrations of alkyl groups. It confirms the presence of an organic coating but strong overlapping signals from Fe_3_O_4_ and SiO_2_ prevent observation of peaks attributed to functionalized dextran. Therefore, the FTIR spectra of dextran and dextran-NH (*or* O)-TEMPO were also compared. In the spectrum of dextran, the broad signal at 3411 cm^−1^ is assigned to –OH stretching vibrations of polysaccharide. The signals at 2935 cm^−1^ (–CH stretching) and 1436 cm^−1^ (H-C-OH deformation) are characteristic of C-H bonds. The bands at 1165, 1118 and 1021 cm^−1^ correspond to vibration of C-O-C bond and glycosidic bridge, C-O vibration and chain flexibility respectively. The glucopyranose ring deformation is confirmed by the presence of characteristic bands at 917, 853 and 769 cm^−1^. Additional bands at 1577 (1584) cm^−1^ in the spectra of dextran-NH (*or* O)-TEMPO correspond to the stretching mode of carbonyl. The signals related to the free radical are located at 1262 (C-N group in TEMPO) and 1365 cm^−1^ (N-O free radical in TEMPO).

The crystallinity of the magnetic materials was investigated by X-ray diffraction (XRD). The samples are characterized by the presence of six diffraction peaks of Fe_3_O_4_ at *2Θ* 30.3°, 35.6°, 43.3°, 53.7°, 57.2° and 62.9° corresponding to the crystal planes of (220), (311), (400), (422), (511) and (440) respectively. The crystalline phase is stable during coating with silica and dextran because the same diffraction peaks are observed in XRD patterns of bare and coated Fe_3_O_4_. The only visible change compared to bare Fe_3_O_4_, is additional broad halo at *2Θ* 22° observed for Fe_3_O_4_@SiO_2_ (Fig. S2). It is a typical feature of amorphous SiO_2_, while crystalline SiO_2_ phase is not found. In the XRD patterns of dextran coated materials, the peaks showed no visible change in relation to Fe_3_O_4_@SiO_2_ and no dextran phase was found. This result is in agreement with literature reports on dextran coated Fe_3_O_4_ nanoparticles^33^. According to transmission electron microscope (TEM) images, the average dimension of bare Fe_3_O_4_ nanoparticles was in 20–25 nm range, while the coated ones were characterized by a larger size (not exceeding 50 nm).

The composition of Fe_3_O_4_ and Fe_3_O_4_@SiO_2_ was investigated by energy dispersive X-ray (EDX) spectroscopy. EDX pattern of bare magnetite shows the presence of Fe and O elements demonstrating the purity of iron(II, III) oxide nanoparticles. Fe_3_O_4_@SiO_2_ contains also Si peak as en effect of silica coating. Additional Cu and C peaks originate from the carbon copper grid (Fig. S3).

### ESR spectra of studied solutions

**Figure 1:**
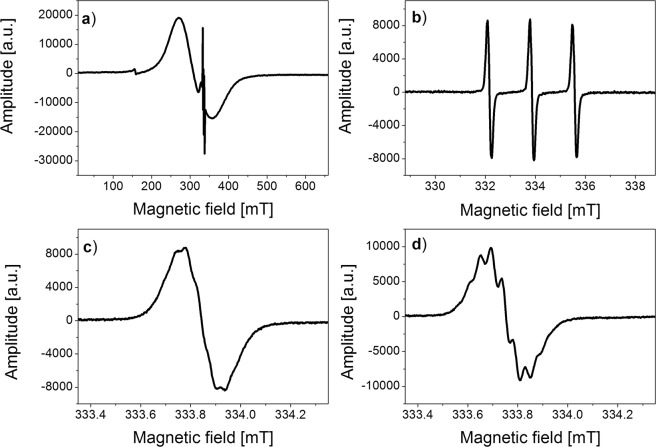
ESR spectra of spin label (Table 1) recorded at 297 K: (**a**) attached to magnetic nanoparticles (wide range of magnetic field, 650 mT), (**b**) narrow range of magnetic field (10 mT) in water, (**c**) central line of triplet shown in Fig. 1b in water (1 mT), (**d**) central line of triplet shown in Fig. 1b with yeast (1 mT).

ESR spectra of the nanoparticles solutions at room temperature showed a typical two-component structure (Fig. 1). The signal from the magnetic core was visible in the whole spectral range (650 mT) (Fig. 1a), while in the narrow spectral range (10 mT) (Fig. 1b) - the one characteristic for spin labels in the form of three hyperfine lines formed as a result of the hyperfine interaction of unpaired electron spin (S = 1/2) with a nitrogen atom nucleus (I_N_ = 1).

The studies of the nanoparticles functionalized with various spin labels carried out at room temperature showed poor differentiation of ESR spectral structure and spectroscopic parameters (spectroscopic splitting factor g, hyperfine splitting constant A, spectral structure, correlation times etc.) regardless of the compound attached to the magnetic core. The differences were only observed for the so-called superhyperfine structure or hyperfine interaction of an unpaired electron with further nuclei of hydrogen atoms in solutions of nanoparticles mixed with yeast cells, aqueous solutions of functionalized nanoparticles and various types of spin labels: TEMPO (2,2,6,6-Tetramethylpiperidine-1-oxyl), TEMPOL, 4-Amino-TEMPO and 4-Carboxy-TEMPO (4-Carboxy-2,2,6,6-tetramethylpiperidine -1-oxyl), (Fig. 1c,d). The superhyperfine structure was better defined for the compounds with functional groups (-OH, -NH_2_, -COOH), especially in solutions with yeast cells (compare Fig. 1c,d).

**Figure 2:**
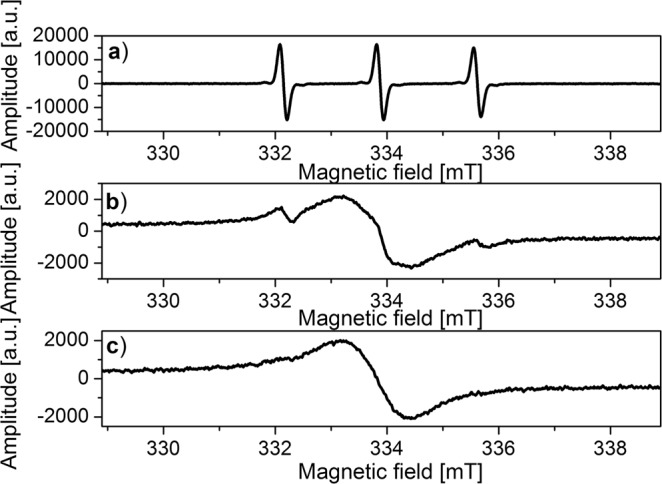
ESR spectra of TEMPO in water recorded at various temperatures: (**a**) 276 K, (**b**) 260 K, (**c**) 250 K.

**Figure 3:**
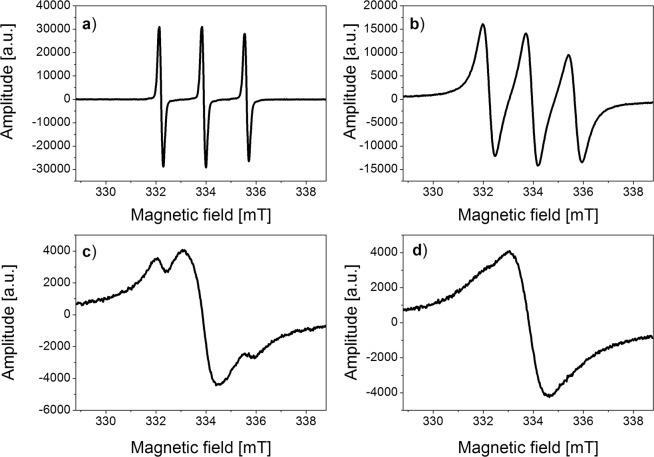
ESR spectra of TEMPOL in water recorded at various temperatures: (**a**) 276 K, (**b**) 250 K, (**c**) 240 K, (**d**) 230 K.

**Figure 4:**
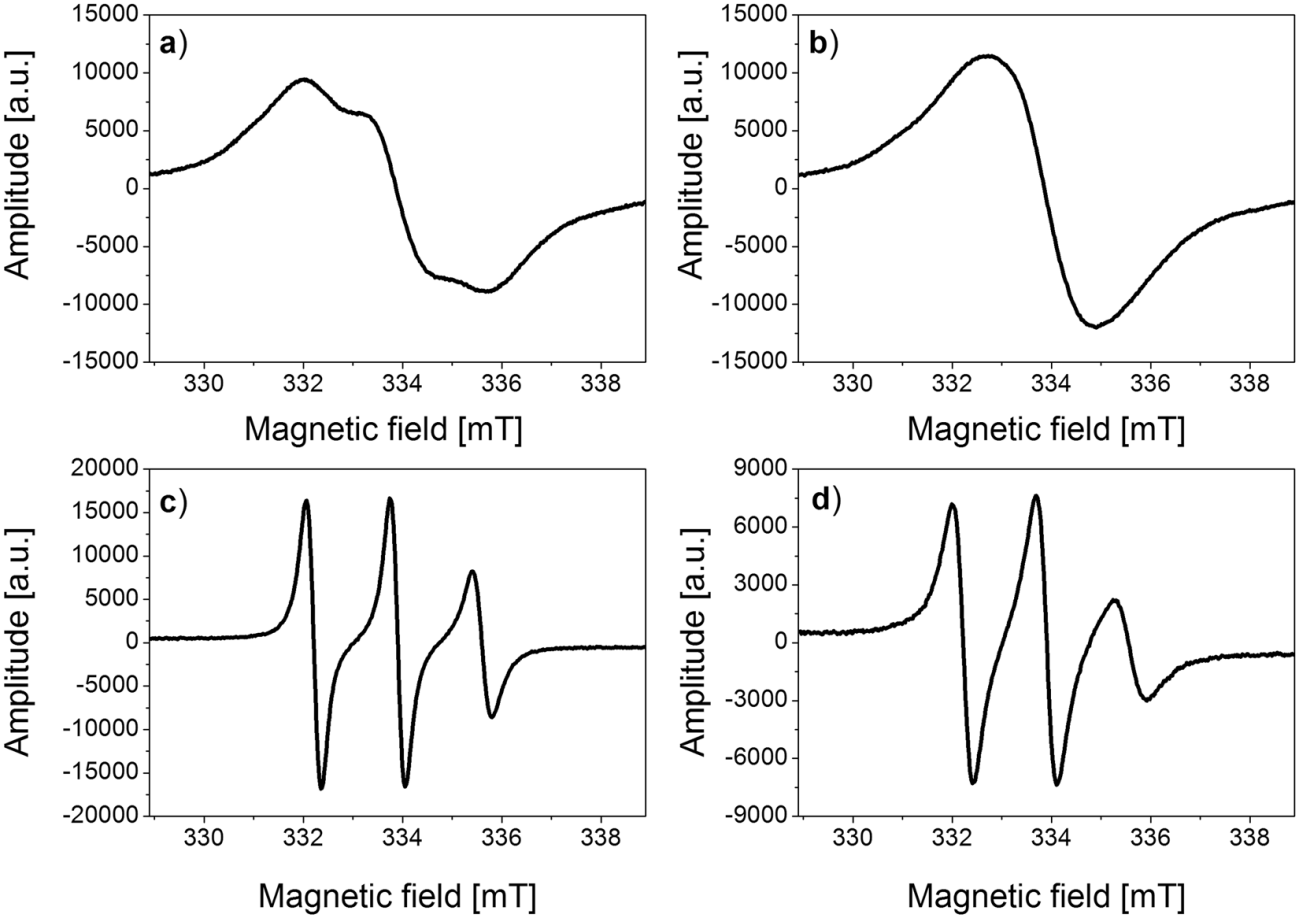
ESR spectra of Fe_3_O_4_@SiO_2_@ Dextran-O-TEMPO: (**a**) in water at 250 K, (**b**) in water at 240 K, (**c**) with yeast at 250 K, (**d**) with yeast at 240 K.

Therefore, in order to diversify the ESR spectra depending on the environment and to visualize their interaction with yeast cells, temperature measurements for the solutions of spin labels, spin labels attached to nanoparticles and the solutions of nanoparticles with yeast cells were performed (Figs. 2–4). Yeast cells with spin labels and with nanoparticles were always incubated at 37 °C. ESR measurements of incubated samples were carried out at low temperatures, by slowing down the molecular dynamics of the nanoparticles, which should result in differentiation of spectroscopic parameters depending on the environmental conditions. Additionally, it was also observed by optical microscope that the sample freezing during ESR measurement did not cause the rupture of yeast cells membrane.

Initially, changes in the structure and parameters of ESR spectra of spin labels were examined (Figs. 2 and 3).

The changes of the hyperfine structure as a function of temperature for the solutions of nanoparticles functionalized with spin labels with or without yeast cells, differ significantly from those in the solutions of spin labels (see Figs. 3 and 4). ESR spectra of the samples studied are significantly different after incubation with yeast cells (Fig. 4).

### Endocytosis and recombination of spin labels

The temperature 240 K (−33 °C) was found to be optimal for differentiation of ESR spectra of the functionalized nanoparticles solution. On the other hand, at room temperature such an effect could not be obtained because the spectroscopic parameters of spin labels (hyperfine splitting, g-factor and spectral structure) were very similar.

The process of yeast incubation with functionalized nanoparticles and especially the time of this incubation had a decisive impact on the structure of the ESR spectrum.

**Figure 5:**
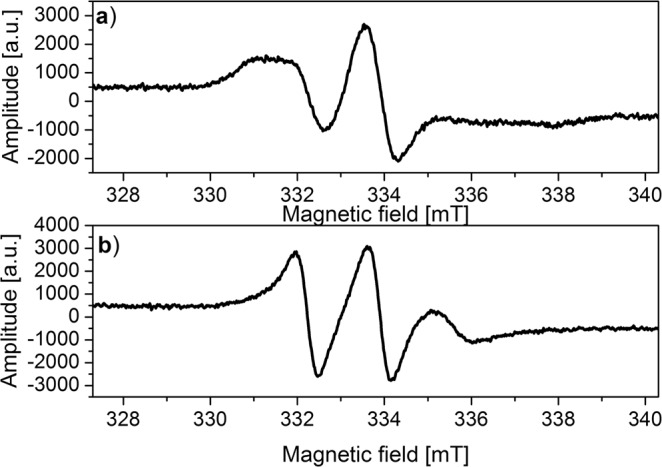
ESR spectra of Fe_3_O_4_@SiO_2_@Dextran-NH-TEMPO in water mixed with yeast cells recorded at 240 K: (**a**) immediately after mixing, (**b**) after 4 h of incubation.

ESR spectra of the nanoparticles studied mixed with yeasts, incubated for about 1 minute (at room temperature), measured at 240 K show a complex structure consisting of radicals differing in molecular dynamics (Fig. 5a).

**Figure 6:**
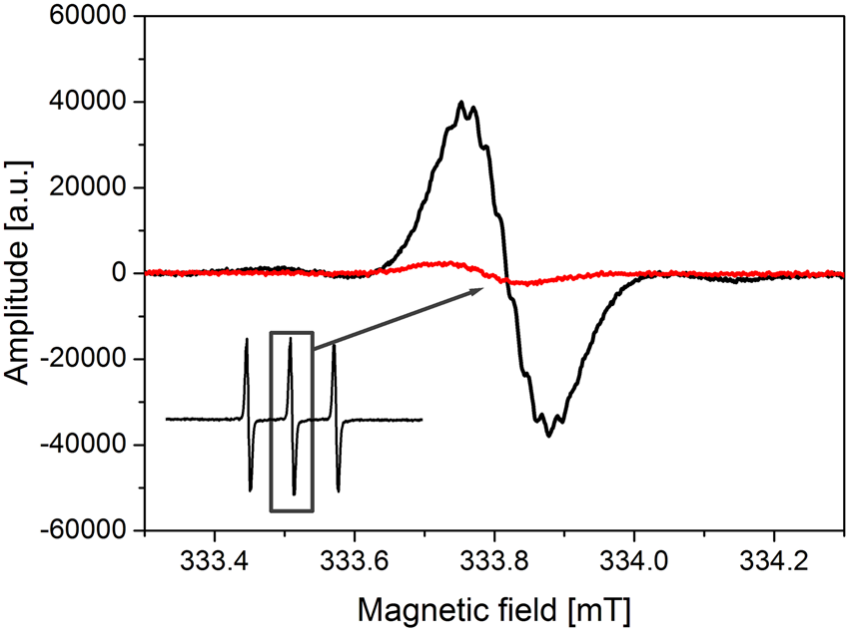
ESR spectra of TEMPO incubated with yeast cells recorded at 295 K: central line of triplet immediately after mixing (black line) and central line of triplet after 60 minutes (red line).

Incubation time had an impact not only on the structure of ESR spectra but also on reducing the intensity of ESR signals derived from spin labels. This allowed the determination of the recombination rate of spin labels during incubation. An example of the rate of recombination of incubated solution yeast-TEMPOL was shown in Fig. 6.

**Figure 7:**
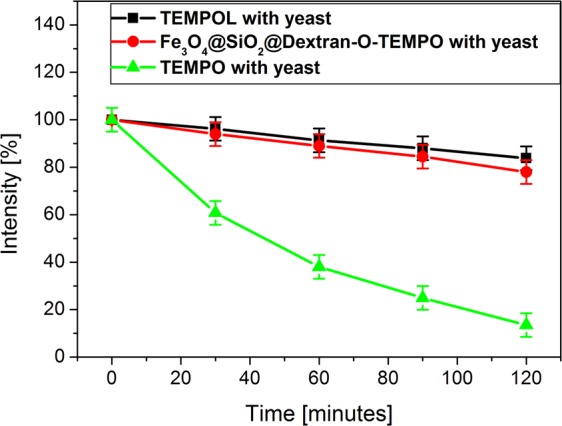
The differences observed in the intensities of ESR signals in time for three studied solutions: yeast cells with TEMPOL, yeast cells with Fe_3_O_4_@SiO_2_@Dextran-O-TEMPO and yeast cells with TEMPO (error bars: the error of ESR signal intensity was taken as ±5%).

Figure 7 presents the differences observed in the intensities of ESR signals in time of incubation for three studied solutions.

### Confocal microscope pictures

**Figure 8:**
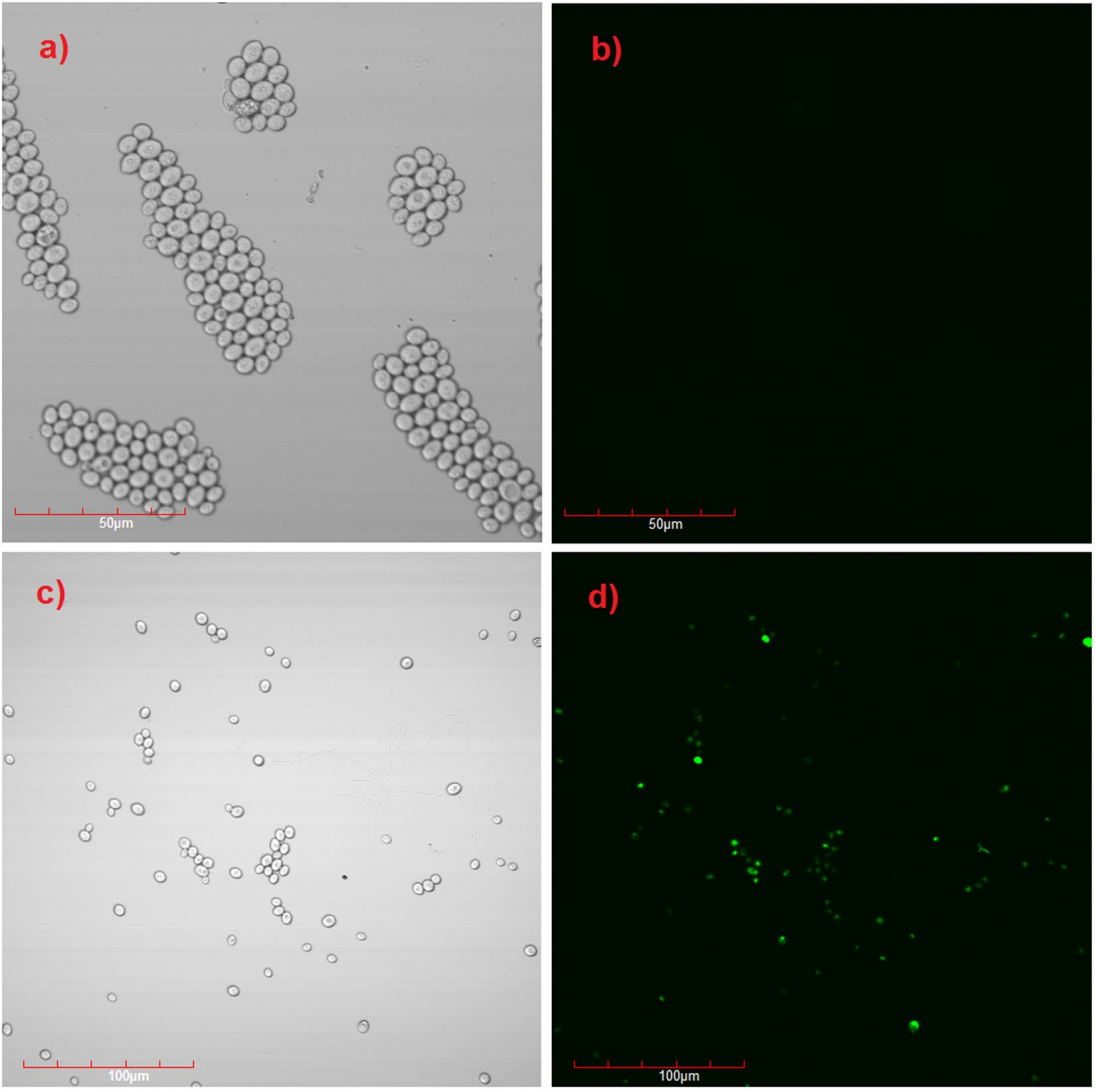
Confocal fluorescent imaging of FITC labeled nanoparticles in yeast cells: (**a,b**) yeast cells without nanoparticles, (**c,d**) yeast cells with Fe_3_O_4_@SiO_2_@FITC nanoparticles.

In order to confirm the endocytosis process of functionalized magnetic nanoparticles into yeast cells, magnetic nanoparticles containing fluorescein (Fluorescein isothiocyanate, FTIC), Fe_3_O_4_@SiO_2_@FITC nanoparticles were used. The pictures taken from confocal microscope (Fig. 8) prove that pure yeast cells (Fig. 8a,b) did not show fluorescence, while the ones incubated previously with Fe_3_O_4_@SiO_2_@FITC nanoparticles gave an opposite effect (Fig. 8c,d). The results confirm that the nanoparticles entered the cells.

## Discussion

Figure 1 illustrates that the superhyperfine structure is better enhanced for solution Fe_3_O_4_@SiO_2_@Dextran-NH-TEMPO – yeast cells (Fig. 1d). The experiments performed at low ambient O_2_ level confirmed no influence of oxygen level decrease during yeast fermentation on better resolution of ESR line in the sample Fe_3_O_4_@SiO_2_@Dextran-NH-TEMPO with yeast cells.

The values of spectroscopic parameters for all samples studied are approximately the same and the superhyperfine structure is best visible for Fe_3_O_4_@SiO_2_@Dextran-NH-TEMPO – yeast cells solution. The parameters were found to be: spectroscopic splitting factor g = 2.168 and the line width ΔH = 87 mT for magnetic core, while for spin labels - spectroscopic splitting factor g = 2.0058, hyperfine splitting constant for a triplet A = 1.71 mT and superhyperfine splitting constant A_s_ = 0.04 mT.

These studies allowed to evaluate the interaction of nanoparticles with cells and therefore the process of endocytosis, as well as the interaction of spin labels attached to nanoparticles with metabolic processes in cells.

A significant decrease in TEMPOL intensity was observed in TEMPOL – yeast solution. At the beginning of incubation at 37 °C the signal was significant and after 60 minutes the intensity of the signal was about 6% of the initial intensity (Fig. 6). It proves that the process of endocytosis and cellular metabolism were very intensive in this case. Moreover, the rate of endocytosis of the nanoparticles functionalized with TEMPOL and yeast is much slower compared to the one of unbound TEMPOL – yeast solution, hence endocytosis for free TEMPOL molecules is much faster.

The recombination process of TEMPOL affects rate of endocytosis and penetration of a spin label into the cell, as well as rate and mechanism of cellular metabolism. It results in reduction of a spin label and structure of ESR spectra.

The exemplary spectra (Figs. 2 and 3) for the solutions of free TEMPO and TEMPOL molecules show the broadening of three components of the hyperfine spectrum as the temperature of the solution decreases. In temperature 240 K/230 K the hyperfine structure disappears and is replaced by a single line (Figs. 2c and 3d).

The changes observed in the structure of the ESR spectra after temperature decreasing, result from broadening of the spectral components as an effect of dipole interactions. Finally, the exchange interactions between the spins of unpaired electron begin to dominate which ultimately leads to the disappearance of the hyperfine structure and the appearance of a single line^34–37^. Similar behavior was observed in literature for the spectra of different spin labels at room temperature in the presence of CrO_x_ compounds^34–37^. The presence of CrO_x_ in the solution results in broadening of the components of the hyperfine spectrum of spin labels and acts as EPR line broadening. CrO_x_ does not pass through the cell membranes. Therefore the changes of the hyperfine structure of spin labels, caused by its interaction with CrO_x_ in the solution containing cells, indicate that the spin labels did not enter the cell and remain in the solution outside the cell. The reduction of the rotational dynamics of the spin labels, caused by the presence of CrO_x_, and the increase of the Heisenberg exchange interaction result in initial expanding and followed by narrowing of individual hyperfine structure triplet lines. It finally leads to the disappearance of this structure and the appearance of a single line. It should be emphasized that this broadening of the spectrum was induced artificially by adding CrO_x_ to the cells solution and it proved the lack of TEMPO penetration inside the cells, i.e. the absence or very slow endocytosis. This suggests that such behavior of the spin labels spectra in the nanoparticles-cells solution at low temperatures would indicate that the nanoparticles do not interact with the cells in the process of endocytosis and behave similarly to pure solution without cells.

In the present study, the broadening of the hyperfine components of the ESR spectrum with decreasing temperature is only observed for solutions of unbound spin labels. At temperatures below 240 K, the exchange interactions between the spin label molecules become a significant factor that affects the narrowing of the entire spectrum. This dominance of exchange interactions is confirmed by the overall width of the spectrum of a single line ΔH = 1.57 mT, which is much smaller than the hyperfine splitting constant of the triplet A = 1.71 mT (Fig. 3).

The spectral structure (Figs. 2–4) shows that the molecular dynamics mechanisms are different for the solutions of unbound spin labels, functionalized nanoparticles without and the ones with yeast cells. As an example, the temperature changes for the solution of free TEMPOL and the one attached to the nanoparticles were compared. The structure of the ESR spectrum for both solutions analyzed differ significantly as temperature decreases. There are two phases in the solutions discussed: the first one represented by broadening of three components of TEMPOL and nanoparticles spectra (Figs. 3 and 4) and the second one manifested by domination of Heisenberg exchange interactions between the radicals. In the solution of TEMPOL in 240 K, two types of radicals are distinguished: first one with narrowed spectrum width 1.64 mT after Heisenberg’s exchange interactions, while the others are narrowed with complete splitting of extreme lines around 3.2 mT. On the other hand, the nanoparticles solution in 240 K is characterized by the presence of only one line (width of 2.2 mT). It indicates much weaker interactions in the nanoparticles studied compared to the solution of unbound TEMPOL. In TEMPOL functionalized nanoparticles, the molecules of free radicals are separated from each other and exchange interactions between them are difficult.

The differences observed are caused by extension of correlation time during sample cooling. It confirms strong interaction of the nanoparticles with the yeast cells. It could be explained by penetration of the nanoparticles into the cell, probably to the endosome. The nanoparticles functionalized with TEMPOL should have sufficient freedom of rotation in the endosome at 240 K. The correlation time for these nanoparticles, estimated on the basis of ESR spectrum, at 240 K is 6 × 10^−9^ s.

As it was mentioned earlier, spectroscopic parameters such as hyperfine splitting, g-factor and spectral structure at room temperature did not show significant differences depending on whether the spin labels were attached to the nanoparticle core or were incubated with yeast cells, etc. However, differentiation was only achieved using a reduced temperature. When the measurement temperature of the studied samples was lowered to 240 K, a clear differentiation of ESR spectra parameters of spin labels was obtained (Fig. 5).

Three lines with shorter correlation time (so-called narrow) and three others that correspond to a longer correlation time (so-called broad) (Fig. 5a) were observed just after mixing the nanoparticle solution with yeast cells. After the sample incubation (4 hours) at 37 °C (Fig. 5b), the signals with a long correlation time disappeared, while the ones with a shorter correlation time were still observed. It probably results from different location of the nanoparticles studied. The so-called narrow triplet is derived from the nanoparticles incorporated into cellular organelles (e.g. endosomes), whereas a broad triplet originates from the nanoparticles associated with the cell membrane or the ones present in the non-cellular environment. The above interpretation is confirmed by measurements of the nanoparticles solution mixed at about 275 K (2 °C). In such conditions, very fast endocytosis process is severely slowed down and the double structure of the ESR spectrum is clearly visible.

Thus ESR was found to be a suitable method for evaluation of endocytosis processes. However, a thorough analysis of this fast process requires further work using solutions at low temperatures in order to slow down or stop endocytosis.

The results of research studies on endocytosis of spin label in red blood cells^38^ indicated easy penetration of spin labels into the membrane of the cell and their reduction to a non-radical form in the process of cellular metabolism. Therefore the process of endocytosis and cellular metabolism result in a reduction of ESR signal intensity. Soule *et al.*^38^ showed that after 60 minutes of incubation of red blood cells with TEMPOL, the intensity of the ESR signal from TEMPOL dropped to about 40% of initial concentration. Thus, ESR could determine the kinetics of TEMPOL reduction occurring in the process of endocytosis and cellular metabolism.

Therefore ESR spectra changes were measured for solutions incubated at 37 °C. The zero point on the graph Fig. 7 shows the intensity of ESR signals for the initial moment, after mixing yeast cells and the nanoparticles solutions incubated at 275 K. Such a low temperature stops endocytosis process^39^. The next points on the graph refer to samples incubated at 37 °C.

## Conclusions

The ESR method allows to study the properties of magnetic nanoparticles functionalized with spin labels. Therefore the magnetic core and the dynamics of the attached spin labels can be precisely monitored.

ESR measurement of the samples containing magnetic nanoparticles functionalized with spin labels at 240 K allowed evaluation of their properties and dynamics in various environments. The same measurements at room temperature would be unlikely to carry out due to fast rotation of spin label molecules.

Moreover ESR measurement at 240 K of the samples containing functionalized magnetic nanoparticles with yeast cells allowed evaluation of the endocytosis and redox processes inside the cell, responsible for recombination of spin labels.

## Methods

### Materials

The spin labels used in the studies are presented in Table 1. The free radicals were purchased from Sigma-Aldrich.

**Table 1:** The spin labels (free radicals) studied.

	Compound	Abbreviation	Structure
1	2,2,6,6-Tetramethylpiperidine-1-oxyl	TEMPO	
2	4-Hydroxy-2,2,6,6-tetramethylpiperidine-1-oxyl	TEMPOL	
3	4-Amino-2,2,6,6-tetramethylpiperidine-1-oxyl	4-Amino-TEMPO	
4	4-Carboxy-2,2,6,6-tetramethylpiperidine -1-oxyl	4-Carboxy-TEMPO	

### The functionalized magnetic nanoparticles

#### Synthesis procedure

FeCl_3_·6H_2_O and FeCl_2_·4H_2_O, hexamethylene diisocyanate, tetraethyl orthosilicate (TEOS), Dextran and solvents were purchased from Sigma-Aldrich. Other chemicals were the analytic grade reagents commercially available and used without further purification. Aqueous solutions were prepared with distilled water.

The synthesis process of iron(II,III) oxide (Fe_3_O_4_) nanoparticles coated with dextran bearing chemically bounded 4-Amino-TEMPO (Fe_3_O_4_@SiO_2_@Dextran-NH-TEMPO) consisted of several steps.

The mixture of 15 mL of 0.02 M FeCl_2_ and 0.04 M FeCl_3_ ([Fe^2+^]:[Fe^3+^ = 1:2) was stirred in a flask, and under a nitrogen. This was followed by the addition of 0.6 mL of 28% ammonia. Upon sonication for 10 min, the reaction mixture was heated to 80 °C for 60 min. The product was magnetically separated from the solution and washed until the solution reached neutral pH. In the next process, magnetic silica nanoparticles, Fe_3_O_4_@SiO_2_, were prepared according to the Ströber method^40^. This synthesis step has already been successfully used to obtain magnetic nanoparticles for a different purpose^41^. First, tetraethyl orthosilicate (TEOS) was dissolved in ethanol (0.1 mL TEOS per 1 mL of ethanol). TEOS solution (15 mL per 1 g of Fe_3_O_4_) was slowly added to a stable suspension of Fe_3_O_4_ (pH 11 adjusted by ammonia) and then the mixture was stirred overnight. The product Fe_3_O_4_@SiO_2_ was magnetically collected, washed several times with water and finally dried at 60 °C.

Dextran functionalized by 4-Amino-TEMPO (Dextran-NH-TEMPO) was obtained as follows: a solution of 4-amino-2,2,6,6-tetramethylpiperidine-N-oxyl (4-Amino-TEMPO) (0.171 g) in acetone was added to a solution of hexamethylene diisocyanate (HDIS) (0.168 g) in acetone ([HDIS]: [4-amino-TEMPO] = 1: 1). After the addition was completed, the solution was stirred for 3 hours, and then dextran (0.120 g) was added. The system was stirred for 12 h.

Finally, Dextran-NH-TEMPO was used to cover and stabilize the magnetite particles. For this purpose the solvent from Dextran-NH-TEMPO solution was evaporated and 0.100 g of Fe_3_O_4_@SiO_2_ in water was added. The mixture was intensively stirred for 1 hour and a stable product Fe_3_O_4_@SiO_2_@Dextran-NH-TEMPO in water was obtained.

Synthesis of Fe_3_O_4_@SiO_2_@Dextran-O-TEMPO was analogous whereby instead of 4-Amino-TEMPO, TEMPOL (O.172 g) was used.

**Figure 9:**
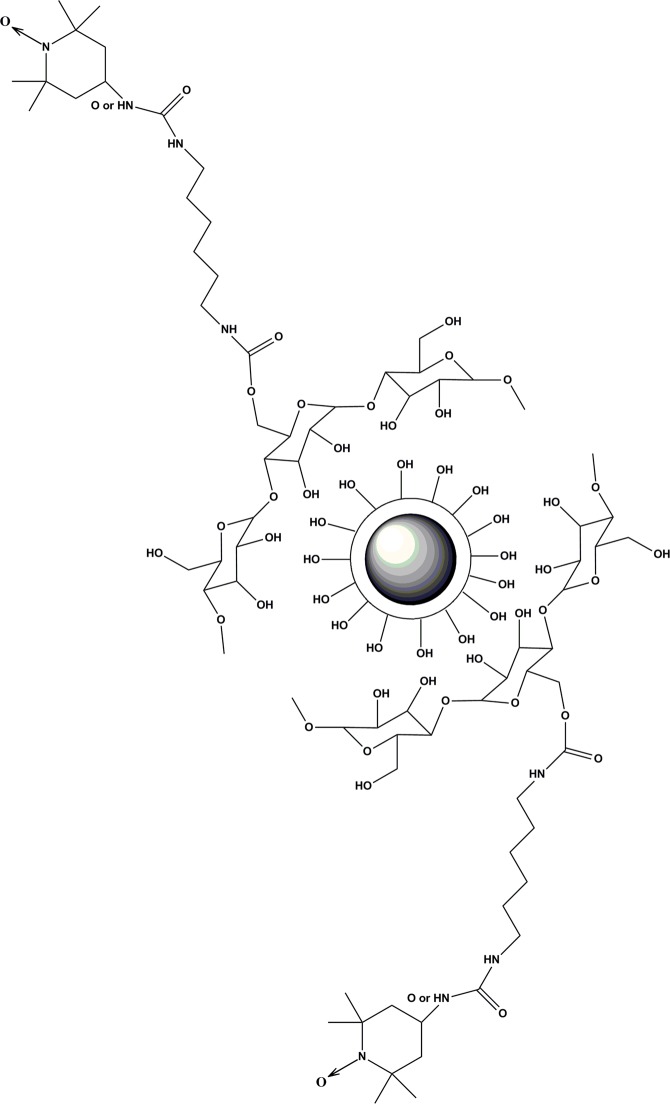
The structure of Fe_3_O_4_@SiO_2_@Dextran-NH-TEMPO and Fe_3_O_4_@SiO_2_@ Dextran-O-TEMPO.

The structures of Fe_3_O_4_@SiO_2_@Dextran-NH (or O)-TEMPO are presented in Fig. 9.

### Synthesis of Fe_3_O_4_@SiO_2_@FITC

Fluorescent labeling of Fe_3_O_4_@SiO_2_ with FITC (Fluorescein isothiocyanate) were synthesized in order to make confocal microscope experiment. The synthesis was carried out according to the procedures described by Yang *et al*.^42^ and Chekina *et al*.^43^. Initially silanization of Fe_3_O_4_ surfaces using tetraethyl orthosilicate was carried out and Fe_3_O_4_@SiO_2_ was obtained. Then it was followed by a reaction with FITC. For this purpose, 0.02 g of FITC dissolved in acetone (10 mL) was added to a suspension containing Fe_3_O_4_@SiO_2_ (0.1 g) in acetone (10 mL). The resulting mixture was stirred and heated at constant temperature (40 °C,12 hours). The Fe_3_O_4_@SiO_2_@FITC were centrifuged, washed several times with the solvent used in the synthesis process, and finally dried at 40 °C. The Fe_3_O_4_@SiO_2_@FITC showed fluorescence characteristic of fluorescein.

### Characterization methods

The magnetic nanoparticles were characterized using several conventional methods^41,44^. An IFS 66 v/s Fourier transform infrared (FTIR) spectrophotometer from Brucker (USA) was used to obtain infrared spectra. The samples were first powdered and the spectra were recorded in the 400–4000 cm^−1^ range. The FTIR spectrophotometer was equipped with MCT detector (125 scans, resolution 2 cm^−1^). Bruker AXS D8 Advance powder diffractometer (Germany) was used for collecting of X-ray diffraction (XRD) patterns. The diffractometer was equipped with Johansson monochromator (λCu K_α1_ = 1.5406 Å). Transmission electron microscope (TEM) images of the samples were recorded on a Hitachi HT7700 microscope (Japan), while EDX patterns were obtained using energy dispersive X-ray (EDX) spectroscopy.

### Yeast cells preparation

Bakery yeast (*Saccharomyces cerevisiae*) (1 g) were diluted in water (50 ml) with addition of sugar (0.3 g) and incubated at 37 °C for one hour.

Water solutions of functionalized magnetite nanoparticles mixed with yeast cells were also incubated during a few hours under the analogous conditions and studied at specified time intervals using the ESR method.

### ESR measurements

Similarly to previous studies^29–32^ the electron spin resonance (ESR) measurements were made on an X-band Bruker EMX –10 spectrometer with 100 kHz magnetic field second modulation frequency. Low temperatures, at which the ESR spectra were recorded, were controlled by a Bruker temperature control system ER 4131VT. The ESR spectra were recorded in a magnetic field sweep range of 650 mT, 10 mT and 1 mT. For the ESR spectra characteristic spectroscopic parameters were determined: g-spectroscopic splitting factor value, peak-to-peak line width (ΔH) and hyperfine splitting constant (A) with the accuracy of ±0.0005, ±0.5 mT and ±0.5 mT, respectively.

The ESR spectra of pure spin labels solutions were investigated: TEMPO, TEMPOL, 4-Amino-TEMPO and 4-Carboxy-TEMPO, spin labels solutions with yeast cells, magnetic nanoparticles solutions with attached spin labels and solutions of magnetic nanoparticles functionalized with spin labels mixed with cells yeast.

ESR method provides information about samples of nanoparticles functionalized with spin labels, which relate to a magnetic core, a structure and dynamics of the surface of nanoparticles as well as their interaction with cells.

### Confocal microscope

Confocal microscope Olympus FV1200 with an excitation laser with a wavelength of 488 nm was used to confirm the endocytosis process. The pictures were taken after one hour of incubation Fe_3_O_4_@SiO_2_@FITC with yeast cells at 37 °C.

## Supplementary information

Supplementary Information

## Data Availability

Data supporting the result of our study are available from the corresponding author upon request.
