# Supplementary material for: ESR as a monitoring method of the interactions between TEMPO-functionalized magnetic nanoparticles and yeast cells
Source: Sci Rep. 2019 Dec 10;9:18733. doi: 10.1038/s41598-019-55335-z (PMC6904674; doi:10.1038/s41598-019-55335-z)
Supplement: Supplementary file 1 — Supplementary Information [file 41598_2019_55335_MOESM1_ESM.pdf]

Supplementary information

ESR as a monitoring method of the interactions between TEMPO-functionalized magnetic nanoparticles and yeast cells

Ryszard Krzyminiewski[1], Bernadeta Dobosz[1*], Grzegorz Schroeder[2], Joanna Kurczewska[2]

[1]Medical Physics Division, Faculty of Physics, Adam Mickiewicz University in Poznań, Uniwersytetu Poznańskiego 2, 61-614 Poznań, Poland

[2]Faculty of Chemistry, Adam Mickiewicz University in Poznań, Uniwersytetu Poznańskiego 8, 61-614 Poznań, Poland

[*]corresponding author: benia@amu.edu.pl

The magnetic nanoparticles studied were characterized using Fourier transform infrared (FTIR) spectroscopy, X-ray diffractometry (XRD), transmission electron microscopy (TEM) and energy dispersive X-ray (EDX) spectroscopy.

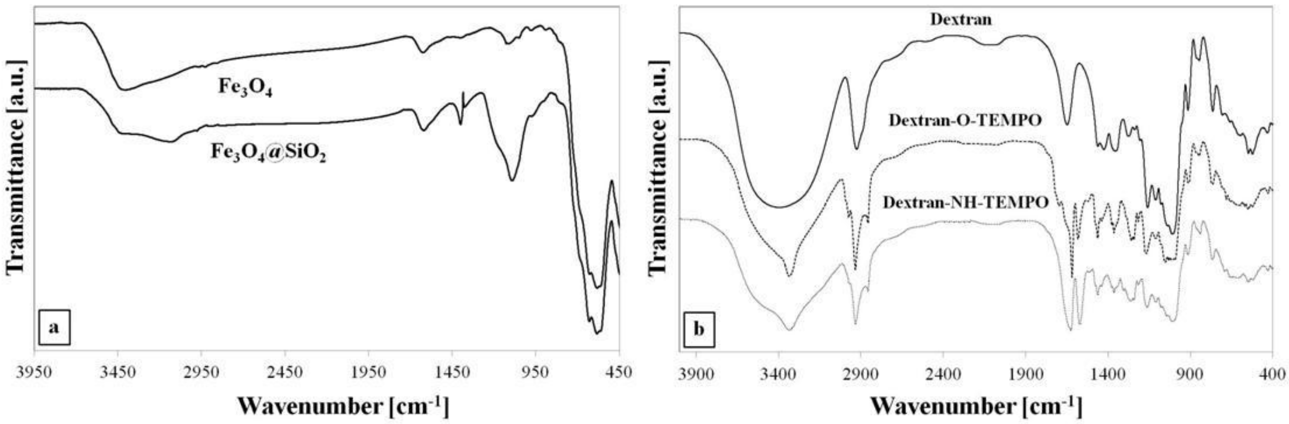

Fig. S1. FTIR spectra of Fe_3O_4 and $Fe_3O_4@SiO_2$ (a), Dextran and Dextran-NH (*or* O)-TEMPO (b)

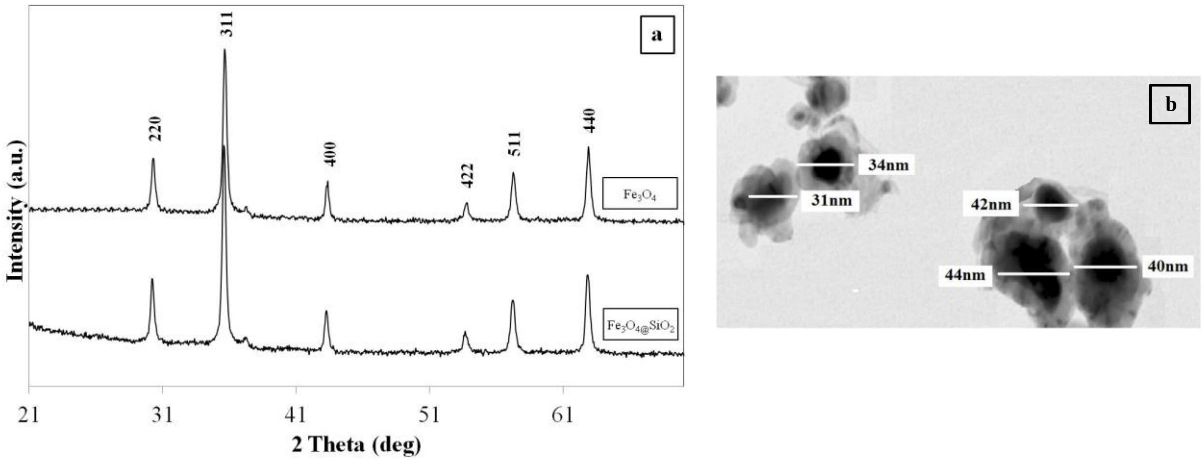

Fig. S2. XRD pattern (a) and TEM image (b) of $Fe_3O_4@SiO_2$

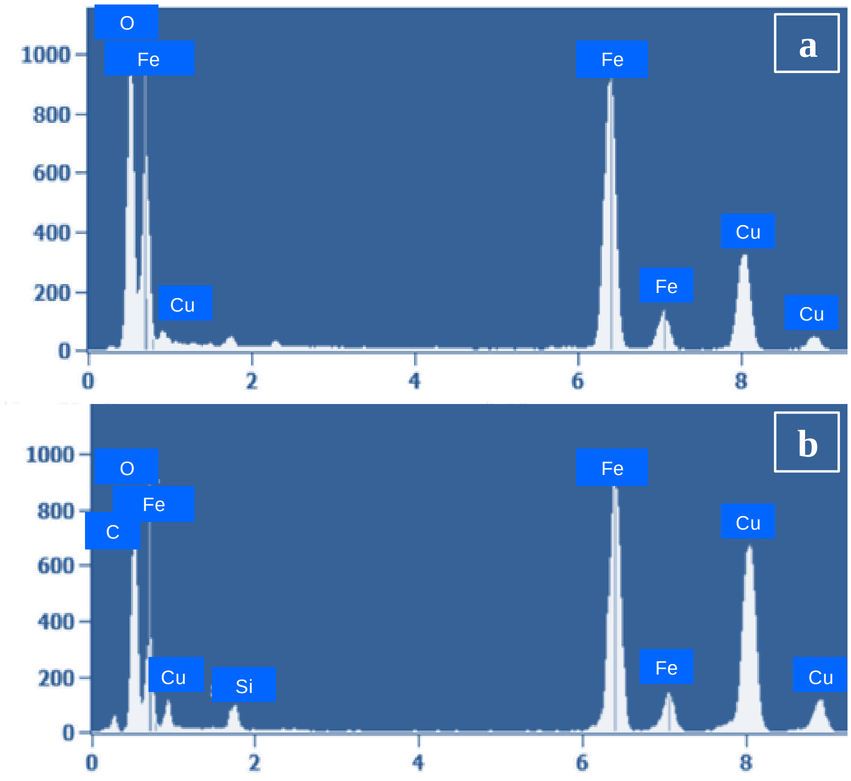

Fig. S3. EDX patterns of Fe_3O_4 (a) and $Fe_3O_4@SiO_2$ (b)
